# Genetic Markers for Coronary Artery Disease

**DOI:** 10.3390/medicina54030036

**Published:** 2018-05-28

**Authors:** Nevena Veljkovic, Bozidarka Zaric, Ilona Djuric, Milan Obradovic, Emina Sudar-Milovanovic, Djordje Radak, Esma R. Isenovic

**Affiliations:** 1 Centre for Multidisciplinary Research and Engineering, Institute of Nuclear Science Vinca, University of Belgrade, 11000 Belgrade, Serbia; nevenav@vin.bg.ac.rs; 2 Laboratory of Radiobiology and Molecular Genetics, Institute of Nuclear Science Vinca, University of Belgrade, 11000 Belgrade, Serbia; bzaric@hotmail.com (B.Z.); obradovicmilan@hotmail.com (M.O.); emma_crash@yahoo.com (E.S.-M.); 3 Department for Endocrinology and Immunoradiology 11080 Zemun, Institute for the Application of Nuclear Energy—INEP, University of Belgrade, 11000 Belgrade, Serbia; ilona.marecko@gmail.com; 4 School of Medicine, Dedinje Cardiovascular Institute, University of Belgrade, 11000 Belgrade, Serbia; radak@ikvbd.com; 5 Faculty of Medicine, University of Belgrade, 11000 Belgrade, Serbia; 6 Serbian Academy of Sciences and Arts, 11000 Belgrade, Serbia

**Keywords:** genetic markers, coronary artery disease, familial hypercholesterolemia, GWAS analysis

## Abstract

Coronary artery disease (CAD) and myocardial infarction (MI) are recognized as leading causes of mortality in developed countries. Although typically associated with behavioral risk factors, such as smoking, sedentary lifestyle, and poor dietary habits, such vascular phenotypes have also long been recognized as being related to genetic background. We review the currently available data concerning genetic markers for CAD in English and non-English articles with English abstracts published between 2003 and 2018. As genetic testing is increasingly available, it may be possible to identify adequate genetic markers representing the risk profile and to use them in a clinical setting.

## 1. Introduction

Coronary artery disease (CAD) and myocardial infarction (MI) are recognized as leading causes of mortality in developed countries [1]. Although typically associated with behavioral risk factors such as smoking, sedentary lifestyle, and poor dietary habits, such vascular phenotypes are also strongly related to genetic background. Based on population and sibling studies, it has been estimated that 40–60% of susceptibility to CAD can be attributed to genetic factors [2,3].

The measurement of genetic markers is nowadays non-invasive, which makes detection of a genetic predisposition for CAD easier. The measuring of such markers is convenient for screening for high-risk individuals very early in life. Also, contrary to circulating biomarkers like cholesterol or triglycerides, genetic markers are not prone to fluctuations. Timely screening could, therefore, allow for better prevention strategies (drug and lifestyle modifications). Given that early modification of risk factors can postpone or prevent the disease [4], it seems reasonable to evaluate genetic variations associated with changeable risk factors such as blood pressure and blood lipid levels.

To create therapies that are effective in CAD treatment, it is of most importance to improve our understanding of numerous genetic, as well as epigenetic cues for the onset and development of heart failure [5,6].

Today several commercial companies offer genetic panels for common diseases, including atherosclerosis. The true utility of these tests, however, is a matter for discussion. 

We anticipate that risk prediction models that incorporate both genetic factors and traditional clinical biomarkers would enable a more reliable estimation of cardiovascular risk and justify preventive measures for individuals at risk. 

## 2. Familial Hypercholesterolemia (FH)

The primary monogenic disease-assuring predisposition to atherosclerosis and CAD is familial hypercholesterolemia (FH). FH is one of the most common genetic disorders, and it is characterized by elevated levels of low-density lipoprotein (LDL)-cholesterol (LDL-C) [7]. This is an inherited disease where a single mutation can lead to a very high risk for atherosclerotic plaque development and premature MI [8]. 

FH is still diagnosed primarily by one’s peripheral blood lipid profile and family history. While genetic screening has not yet been universally adopted, it is recommended, but not mandatory, in the Netherlands, Norway, and United Kingdom [9]. Also, the Simon-Broome criteria and The Dutch Clinical Lipid Network criteria for the diagnosis of FH require a functional genetic mutation as well [10,11]. In the United States, the American Heart Association (AHA) encourages genetic testing [12], while the US Medical Pedigrees with FH to Make Early Diagnoses and Prevent Early Death (MEDPED) has not yet adopted this recommendation [13]. Practice shows that the implementation of strategies for identification of individuals with FH at the population scale has proven difficult, making FH both underdiagnosed and undertreated [11]. Apart from cost and logistical concerns, large genetic screening is additionally complicated by the fact that knowing the mutation status of the individual is not sufficient for diagnosis. Not all carriers of FH mutation manifest severely elevated cholesterol levels [14]. A study [14] showed that 27% of these individuals had normal LDL-C concentrations (suggesting incomplete penetrance of the mutation). On the other hand, among individuals diagnosed with severe hypercholesterolemia, having LDL-C level ≥ 190 mg/dL (4.91 mmol/L), only 2% were carriers of FH mutations [15]. These data imply that monogenic disorders account for a very small portion of diagnosed cases of atherosclerosis-related diseases. The majority of cases are polygenic, resulting from complex interactions among genetic, epigenetic, and environmental factors [16,17,18].

**Figure 1:**
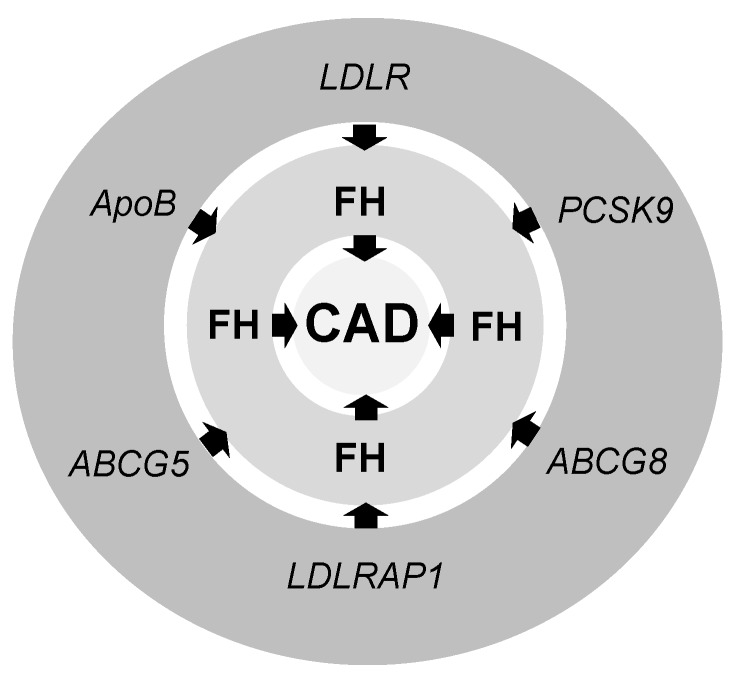
The candidate genes for genetic markers of FH and consequently CAD. ABCG5—ATP-binding cassette sub-family G member 5 (or ABCG8); ApoB—Apolipoprotein B; CAD—coronary artery disease; FH—familial hypercholesterolemia; LDLR—low-density lipoprotein receptor; LDLRAP1—LDLR adaptor protein 1; PCSK9—Proprotein convertase subtilisin/kexin type 9.

The candidate gene approach pinpoints several mutations responsible for FH and consequently CAD (Figure 1): A mutation in the LDL receptor (LDLR), a mutation in apolipoprotein B (ApoB), a gain of function mutation in proprotein convertase subtilisin/kexin type 9 (PCSK9) genes, and null mutations in the genes encoding LDLR adaptor protein 1 (LDLRAP1) and ATP-binding cassette sub-family G (ABCG) member 5 (ABCG5) or member 8 ABCG8 (Figure 1) [19].

## 3. Genetic Markers for CAD

Genetic epidemiologic methods for recognition of candidates for complex traits follow two main approaches. A hypothesis-driven approach explores a potential candidate gene or a pathway with a large effect on disease development, while a hypothesis-free approach relies on population-based studies like genome-wide and rare-variant association studies [20].

The hypothesis-driven approach relies on prior knowledge of the disease aetiology. These studies usually focus on deleterious loss-of-function mutations that follow the Mendelian pattern of inheritance [21,22]. The disadvantage of candidate gene analysis is the lessened possibility for detection of new genetic variants or novel genes. In addition, genes with small or modest effect on disease course can be missed [20,23]. 

A hypothesis-free study design uses large cohorts of unrelated individuals that are genotyped at millions of single nucleotide polymorphisms (SNPs) across the genome. The accomplishments of the Human Genome Project and the HapMap project, combined with the development of large-scale genotyping methods supported by statistical and computational approaches, enabled large-scale Genome-wide association studies (GWAS), in which a large number of genetic variants are investigated in a search for links with the trait of interest [24]. GWAS is extremely successful, making up to 2,000,000 genetic variants available for association analysis with a given phenotype [25]. Additionally, GWAS analysis is unbiased by previous knowledge and is therefore useful for detecting novel unsuspected gene candidates.

More recently, next-generation sequencing (NGS) technologies have also enabled the “rare variant association study” (RVAS). Genetic variants that are too rare to be detected by GWAS are aggregated into subsets, and their frequency is compared between patients and controls [26]. 

## 4. Low-Density Lipoprotein Receptor (LDLR)

In the body, the principal receptor responsible for clearance of LDL-C from blood circulation is hepatic LDLR [27]. LDL-C binds LDLR and forms LDL-C/LDLR complexes that undergo endocytosis within clathrin-coated vesicles [28,29]. After translocation to the cytoplasm, LDL-C separates from LDLR, and it is subject to further degradation, while LDLR rapidly recycles and folds back to the cell surface [28,29]. The mechanism of LDL-C uptake by the LDLR is a very specific process, and it is influenced by various hereditary and environmental factors [27,30]. FH and its consequences [31,32] can be caused by mutations in the LDLR gene. There are several gene mutations of the LDLR that lead to mild or severity FH, such as mutations that affect the synthesis of the LDLR in the endoplasmic reticulum, mutations that disable proper transport of LDLR to the Golgi apparatus, mutations that disable binding of LDL-C to the LDLR, mutations that disable the receptor-ligand complex internationalization, and mutations that disable proper recycling of LDLR [31,32,33]. In addition, the PCSK9 indirectly controls the level of LDL-C in the blood by binding to the epidermal growth factor-like repeat homology domain (EGF-A) of the LDLR in the liver, which leads to endocytosis and LDLR destruction [34].

## 5. Apolipoprotein B (ApoB)

ApoB is an essential structural protein component of all atherogenic or potentially atherogenic lipoprotein particles, including chylomicrons, very-low-density lipoprotein (VLDL), intermediate-density lipoprotein (IDL), LDL, and lipoprotein (a) (Lp(a)) [35,36]. Each of the particles mentioned above contains one molecule of ApoB [35,36]. The concentration of atherogenic particles can be accurately estimated by measuring the plasma level of this apolipoprotein [34]. ApoB remains anchored to the lipoproteins without undergoing any changes [37]. Therefore, an increased plasma ApoB concentration is an important risk factor/predictor of CAD [35,36]. ApoB provides a direct measure of the number of atherogenic lipoprotein particles in circulation. The majority of the total plasma ApoB is bound to LDL, which makes ApoB a good substitute for LDL particle concentration [35,38]. Higher ApoB lipoprotein particles may be less atherogenic than the smaller, denser LDL particles. Therefore, the measurement of the level of ApoB in LDL particles is a better predictor of atherogenesis than the total serum ApoB level, although this is not documented in all published studies [35,39,40,41,42]. ApoB is assumed to be a superior marker for lipoprotein abnormalities [36]. The blood level of ApoB in patients with CVD has been shown to be a better discriminator than HDL-C and LDL-C levels. 

ApoB is present in plasma as two main isoforms, ApoB-48 and ApoB-100 [43]. The Apo-48 is exclusively found in the gut, and the Apo-100 is found in the liver [44]. The intestinal and the hepatic forms of ApoB are encoded by a single gene, which gets transcribed into long mRNA [45,46].

ApoB-100 is a predominant structural apolipoprotein of LDL particles, and it binds to LDLR and mediates hepatic LDL-C uptake. Therefore, it is expected that mutations in ApoB and LDLR affect the level of cholesterol and lead to hypercholesterolemia and the development and progression of CVD [47]. 

## 6. Proprotein Convertase Subtilisin/Kexin Type 9 (PCSK9)

PCSK9 is a crucial modulator of LDLR levels and plasma LDL-C [48]. Deficiency in PCSK9 leads to considerably lowered LDL-C levels in humans and protects against CAD [49,50]. 

In the 1960s and 1970s, it was established that bioactive secretory proteins (hormones and enzymes) initially are synthesized as inactive precursors which are transformed into active products by limited proteolysis [51]. This introduced the concept that conversion of an inactive precursor into the product which fulfils its function is catalyzed by a special group of proteases called proprotein convertase. PCSK9 binds to the LDLR and enables its degradation, which leads to a decrease of LDL-C and an increased risk of atherosclerosis. As a result, PCSK9 emerged as a promising therapeutic strategy for the treatment of hypercholesterolemia and atherosclerosis. 

The gene which encodes for the PCSK9 is located on chromosome 1 [52,53] and encodes for the member of the subtilisin-like proprotein convertase family that activates other proteins, and its coding region is comprised of 13 exons [52]. In 2003, through the protein BLAST program [54], a putative convertase called neural apoptotic-regulated convertase 1 (NARC-1), which belongs to the proteinase L subfamily of subtilases, was identified [55]. At the same time, a research group in Paris (Necker Hospital) studied families with FH, the genetic form of an extremely high level of LDL-C caused by the expression of the gene on the short arm of chromosome 1 [56], which leads to the development of severe CAD, often resulting in premature death.

Most therapeutic approaches to hypercholesterolemia involve cholesterol biosynthesis inhibition and upregulation of LDLR in the liver. The analysis [57] of 1183 patients was conducted and showed that with statin treatment there is a large reduction in LDL-C down to 60 mg/dL (1.55 mmol/L). People with mutations in ApoB and LDLR can develop hypercholesterolemia, as can people with mutations in both ATP-binding cassette transporters (ABCG5 and ABCG8) and the gene called autosomal recessive hypercholesterolemia, which encodes for the LDLR adaptor protein called PCSK9 [58]. Mutations in the PSCK9 were first described in the family of persons who developed FH [30,56]. The link between PCSK9 and cholesterol metabolism was followed by the discovery of selected mutations in the gene and the observation that PCSK9 was regulated by cholesterol [59]. 

## 7. LDLR Adaptor Protein 1 (LDLRAP1) 

Autosomal recessive hypercholesterolemia is a very rare disorder (mostly in Italians) caused by a mutation in the LDLRAP1 gene [60,61]. LDLRAP1 (previously termed autosomal recessive hypercholesterolemia (ARH)) is the protein involved in regulation of proper traffic and recycling processes of LDLR [60,61]. This adapter protein contains a phosphotyrosine binding (PTB) domain that recognizes and binds to a conserved tyrosine phosphorylation motif (Asn-Pro-X-Tyr) where X is any amino acid (NPXY motifs) of membrane receptors, including LDLR [60,61]. Defects in mature LDLRAP1 caused by mutations of that gene lead to incorrect LDL uptake of hepatocytes, resulting in hypercholesterolemia. Unlike patients with a homozygous genotype, clinical presentation in patients with a heterozygous genotype often have a normal level of cholesterol in the circulation [13,61,62].

## 8. Adiponectin

Adiponectin is one of the most abundant adipocyte-derived secretory proteins in human visceral fat tissues. Circulating levels of adiponectin are negatively correlated with the percentage of human visceral fat mass [63]. Adiponectin is a 247-amino-acids-long protein with structural and sequence homology (43%) with tumor necrosis factor-α, and complement protein C1q Adiponectin is composed of three domains: a signal sequence located at the N-terminus, a collagen-like domain, and a globular C terminal domain [64]. In the circulation, adiponectin exists as the hexamer called low molecular weight oligomer (HMW), which is composed of four to six trimmers (the active form of adiponectin) [65]. HMW oligomers are part of the intracellular adiponectin, while within circulation, adiponectin is represented as the low molecular weight oligomers. Circulating adiponectin represents 0.05% of total serum protein [2], and usual concentrations in the circulation are between 2 and 20 µg/mL^−1^. High plasma levels of adiponectin are related to insulin sensitivity in a healthy population [3]. Lower levels of adiponectin are a risk for development of diabetes [66], CAD, and hypertension [67,68]. Adiponectin exerts atheroprotective characteristics and has inverse relations with CAD [6]. Adiponectin modulates the interaction between classical risk factors and atherosclerosis [69]. Levels of adiponectin are lower in patients with cardiovascular diseases, and lowered levels of adiponectin can be a predictor of the development of myocardial infraction [66,67,68]. Adiponectin is a cardioprotective protein, yet its association with the atherosclerotic severity and predictive power for CAD remains controversial in different populations, most likely due to racial/ethnic differences, lifestyles, and environmental factors [70,71,72]. Plasma concentrations of adiponectin and HMW adiponectin might be useful as the early biomarkers of cardiovascular risk in general and also a predictor of adverse cardiovascular events in patients with CAD [70,71].

## 9. C-Reactive Protein (CRP)

C-reactive protein (CRP) is an acute phase protein synthesized in the liver and the vascular endothelium, and it belongs to the family of pentraxins. In atherosclerotic plaques, CRP is present with monocytes and lipoproteins [73]. CRP activates the process of phagocytosis, which clears necrotic tissues in the atherosclerotic plaques and perpetuates inflammatory response [74]. There are indications that persons with no manifestations of vascular disease and elevated CRP have a 3–4 fold increased relative risk of myocardial infarction [75]. In a large meta-analysis on subjects with no history of vascular disease, CRP was connected with a risk of CAD and ischemic stroke [76]. Elevated CRP is associated with an increased risk of CAD events in apparently healthy individuals [77], and its elevated levels are strongly associated with the risk of fatal CAD outcomes. Baseline levels of CRP are elevated in patients with unstable angina and are associated with an unfavorable short-term prognosis. CRP levels might be a valid prognostic marker for differentiation between patients with unstable angina and chronic stable angina; however, they fail to differentiate patients with stable CAD from patients with acute coronary syndrome [78].

In patients with an angiographically evaluated CAD, levels of high-sensitivity CRP (hs-CRP) are significantly higher compared to healthy individuals, and they correlate with the severity and presence of the CAD [79]. There are undoubtedly advantages to hs-CRP measurements for CAD detection and evaluation. CRP is a stable protein, and its levels can be measured at any time of the day without special relevance to the biological clock [74,80].

## 10. Ion Channels

The alternated or disturbed regulation of the coronary blood flow can lead to CAD. Ion channels are key effectors of the regulatory mechanism, and certain variations in genes encoding for ion channel proteins may affect the coronary blood flow [81]. Polymorphisms in ion channel genes are also recognized as contributors to diabetes mellitus, which is one of the most powerful cardiovascular risk factors [82,83]. Evaluation of the clinical impact of these SNPs showed that polymorphism detected in nitric oxide synthase (NOS) 3 gene (NOS3), which encodes for endothelial NOS (eNOS), are correlated with ischemic heart disease [84]. The SNP rs1805124_GG for a sodium channel alpha-subunit gene (SCN5A) of the voltage-gated sodium channel, Nav, is more frequently observed in patients with CAD [84]. SNPs in ATP-sensitive potassium channel (KATP) subunits KCNJ8 (Kir6.1) and ABCC9 (SUR2) [83] might influence the presence of diabetes, and they seem to be involved in ischemic heart disease pathogenesis. However, the mechanisms of this effect are still unclear.

## 11. GWAS Analysis and CAD

To uncover multiple loci spanning the entire genome responsible for the onset of atherosclerosis, large-scale GWAS studies were performed [85,86]. GWAS analysis allows for simultaneous and accurate genotyping of up to 1 million SNPs. For each SNP, individuals with one genotype are compared with individuals with another genotype to assess whether there is a phenotypic difference. GWAS studies require very large samples, which permit detection of alleles with low incremental risk. 

This called for the establishment of large international collaborations in the field of cardiology. The international consortium, CARDIoGRAM (Coronary Artery Disease Genome-Wide Replication and Meta-Analysis), is the biggest such alliance to date, and it has analyzed more than 200,000 cases and control subjects of European ancestry. Apart from sufficient sample size, this collaboration brought together resources and researchers from United States, Canada, United Kingdom, Germany, and Iceland. The group identified 62 loci associated with predisposition to CAD, which were subsequently confirmed in a different population [87].

Interestingly, only 20% of the loci were spotted in proximity to the genes with known roles in the metabolism of LDL or triglyceride-rich lipoproteins (TRLs). An additional 5–10% of the loci regulate vascular tone or platelet aggregation [85]. These findings further confirmed the importance of already recognized contributors, but interestingly, some unsuspecting candidates emerged. Genes involved in focal adhesion/extracellular matrix interaction, transforming growth factor beta (TGF-β) signalling, apoptosis, angiogenesis, and transcriptional processes, whose role is not entirely clear, were significant [88]. Moreover, the spotted SNPs are supposed to have cumulative or synergistic effects because, taken singly, each one of them has a minimal or modest effect. The relative increased risk of each genetic variant for CAD averages only 18% [89]. The genetic risk for CAD seems much more associated with the number of inherited risk variants than with the power of any one genetic variant alone. 

However, the majority of SNPs detected by GWAS are not in coding sequences, and the mechanisms underlying these associations are less than obvious [90]. They may exert functional consequences if they are localized in the promoter regions or through mRNA silencing. This implies that there are more unknown mechanisms contributing to the pathogenesis of CAD than suspected. These genetic variants occur very frequently, about half occurring in 50% of the population and a quarter in more than 75% of the population, whereas they cumulatively explain only 30–40% of CAD heritability [85,91]. 

A series of reports documented that one such SNP is in the 9p21.3 locus [90,92]. Approximately 75% of the European population carries this locus, and it is independent of any conventional risk factor for CAD. 25% of Europeans that carry two copies of this SNP have a 40% increased risk of atherosclerosis [93,94]. The association of this locus with the atherosclerotic phenotype was confirmed in independent studies and among different ethnic groups. The same variants have been linked to both abdominal aortic and intracranial arterial aneurysms, suggesting its possible effects on vascular wall integrity [95]. 

Some evidence indicates that the 9p21 is a part of the long non-coding RNA (lncRNA) antisense noncoding RNA in the INK4 locus (ANRIL), which affects the activity of two nearby cyclin-dependent kinase (CDK) inhibitors, 2A (CDKN2A) and 2B (CDKN2B) [68,69]. Because the 9p21 risk variant is not present in the mouse genome, it is difficult to determine its function, and the precise mechanism underlying the 9p21 association with CAD remains vague.

GWAS analysis showed an association of genetic factors with only a small number of disease phenotypes. The genetic variants have relatively low effect sizes and explain up to 40% of population variation. This leaves the best part of heritability unknown, possibly due to missing gene-environment interactions [96,97]. The findings of Cole et al. [98], that genetic risk for dyslipidemia is positively associated with adiposity, imply the necessity of screening for other lifestyle predictors that may enhance gene variants effects, such as physical activity, smoking, and alcohol intake.

Several groups have made an effort to translate the genetic risk burden identified by GWAS into a single value, a genetic risk score (GRS), which is convenient for patient stratification [99,100]. A GRS depends on both the number of high-risk variants inherited and the log of the odds interval previously determined. These studies suggest that GRS is independent and more accurate than traditional risk factors. A prospective trial showed individuals with a high GRS had a 91% greater risk of cardiac events [89]. The study of Mega et al. [101] demonstrated that a high GRS is not only associated with the incident of CAD events but also foresaw recurrent disease. A subsequent study analyzed 23 additional SNPs and showed even more improved discrimination and reclassification than the study of Tada et al. [102]. Individuals with high GRS had a 2.4-fold greater risk than those with low GRS. Other studies that included even more loci report further enhancement of the power of predicting cardiac events [103,104].

However, the high cost of genotyping, and the widespread use of Framingham or American College of Cardiology/American Heart Association (ACC/AHA) risk scores that already perform quite well, makes it difficult to demonstrate sufficient improvement in patient management [105,106]. 

## 12. Conclusions

Given the complex genetic background of vascular diseases, including family history in the initial medical evaluation appears reasonable. In cases where first-degree relatives are affected and inheritance patterns hint at monogenic disorders, investigational clinical studies show that genetic testing is justified. In other cases, however, testing for genetic factors still offers little advantage over the examination of traditional risk factors.
